# Two wild female bonobos adopted infants from a different social group at Wamba

**DOI:** 10.1038/s41598-021-83667-2

**Published:** 2021-03-18

**Authors:** Nahoko Tokuyama, Kazuya Toda, Marie-Laure Poiret, Bahanande Iyokango, Batuafe Bakaa, Shintaro Ishizuka

**Affiliations:** 1 Primate Research Institute, Kyoto University, Kanrin 41, Inuyama, Aichi 484-8506 Japan; 2 Wildlife Research Center, Kyoto University, Kyoto, Japan; 3 Department of Psychology, Durham University, Durham, UK; 4 Research Center for Ecology and Forestry, Mabali, Mbandaka, Democratic Republic of the Congo

**Keywords:** Animal behaviour, Anthropology, Evolution, Zoology

## Abstract

Adoption, the act of taking another individual’s offspring and treating it as one’s own, is rare but widely observed in various mammal species and may increase the survival of adoptees. Adoption may also benefit adoptive mothers, for example they might care for close kin to gain indirect fitness or to learn caregiving behaviours. Here, we report two cases of a wild bonobo adopting an infant from a different social group, the first report of cross-group adoption in great apes. In one case, the adoptive mother was already a mother of two dependent offspring. In the other case, the adoptive mother was an old parous female whose own offspring had already emigrated into a different social group. The adoptive mothers provided various maternal care to the adoptees, such as carrying, grooming, nursing, and sharing food. No aggression was observed by group members towards the out-group adoptees. In both cases, adoptees had no maternal kin-relationship with their adoptive mothers. Both adoptive mothers already had experience of rearing their own offspring. Instead, these cases of adoption may have been driven by other evolutionary adaptive traits of bonobos, such as their strong attraction to infants and high tolerance towards immatures and out-group individuals.

## Introduction

Mothers play a pivotal role in infant survival across mammals. They provide milk to feed infant(s), protection, hygiene, transportation, food, and other forms of care^1^. Maternal care also plays a crucial role in the development of an infant’s social, emotional, and cognitive skills^2–4^. In social species, individuals other than mothers may also care for immatures (alloparenting), and such behaviour may incur some cost to the alloparents^5,6^.

Adoption, when an individual provides exclusive maternal care to another individual’s offspring, is considered to be the costliest type of alloparenting because the adoptive mother pays a similar cost as a mother would pay for her biological offspring. Although rare within a species, this behavior has been observed across various different mammal species^5,7–14^. Adoption is typically observed when the biological mother dies and may increase the orphan’s chance of survival^5,7,8^. It may also bring benefits to the adoptive mothers, who can be female or male and of various ages^5^, which could outweigh the cost of providing alloparental care^5^. Adoption is often observed among close kin and explained by kin selection, where infant survival increases the indirect fitness of the adoptive mothers^5,8,9,15,16^. Adoption may also increase direct fitness; for example, the Learning-to-Mother hypothesis proposes that alloparenting is a way for individuals to learn caregiving behaviours, thus increasing the survival chances of their future offspring^5,17,18^. An adoptive mother’s social status may also increase if the adoptee becomes a social ally, and having the adoptee may increase her reputation in the group^5,19,20^. In chimpanzees, kin or non-kin individuals which had a close affiliative relationship with a deceased mother tended to provide alloparental care to her orphan, so constructing and maintaining social bonds with group members likely benefits mothers^7,8,21^.

In some cases, including those among humans, adoption cannot be explained purely by benefits that adoptive mothers receive and may be altruistic and emotional^7,21–24^. Humans adopt unrelated individuals without pre-existing social relationships, and after acquiring high mobility between regions, it has become more common to adopt cross-culturally^24,25^. Adoption beyond one’s own social group is extremely rare in other group-living animals, especially in non-human primates where most species form stable groups and identify group members from out-group individuals using visual, auditory, and olfactory cues^26–29^. There are only two reported cases of non-human primates adopting an out-group infant: one in black-fronted titi monkeys which form monogamous family groups^30^ and the other in Angola black-and-white colobus monkeys which live in one-male multi-female groups^31^.

Bonobos (*Pan paniscus*), along with chimpanzees, are humans’ nearest evolutionary relatives. They form multi-male, multi-female social groups with male philopatry, where males remain in their natal group throughout their life and females typically emigrate before reaching sexual maturation^32,33^. Inter-group relationships in bonobos can be tolerant; separate groups sometimes associate for a few days^34–36^. When an inter-group association ends, individuals usually separate back into their original group, except for nulliparous immigrant females. Although bonobos of different groups can be tolerant of each other, they (especially males) may act aggressively and form coalitions to attack individuals of other groups, suggesting that inter-group competition exists^37^. However, severe aggression within and between groups, including infanticide, has never been reported in bonobos^38^. They are tolerant of immature individuals, and group members of all sex and age categories, especially nulliparous females, engage in alloparenting behavior^18,39^.

Bonobo physical and social developmental stages are largely similar to those of chimpanzees; infants are carried by their mothers until weaning at around 4–5 years old, and they maintain close associations beyond this period^40–42^. In chimpanzees, the death of a mother negatively affects her offspring’s survival before and after weaning, especially when orphans are younger than 5 years old^40,43,44^. Physical, cognitive, emotional, and social development can be impaired as well in both bonobos and chimpanzees^40,45–48^. Even after weaning, the loss of a mother negatively affects males’ reproductive success^49,50^. Only one case of adoption has been reported in wild bonobos: a 4 year old male infant was adopted by his older brother after their mother’s death and survived for > 2 years^51^.

**Figure 1:**
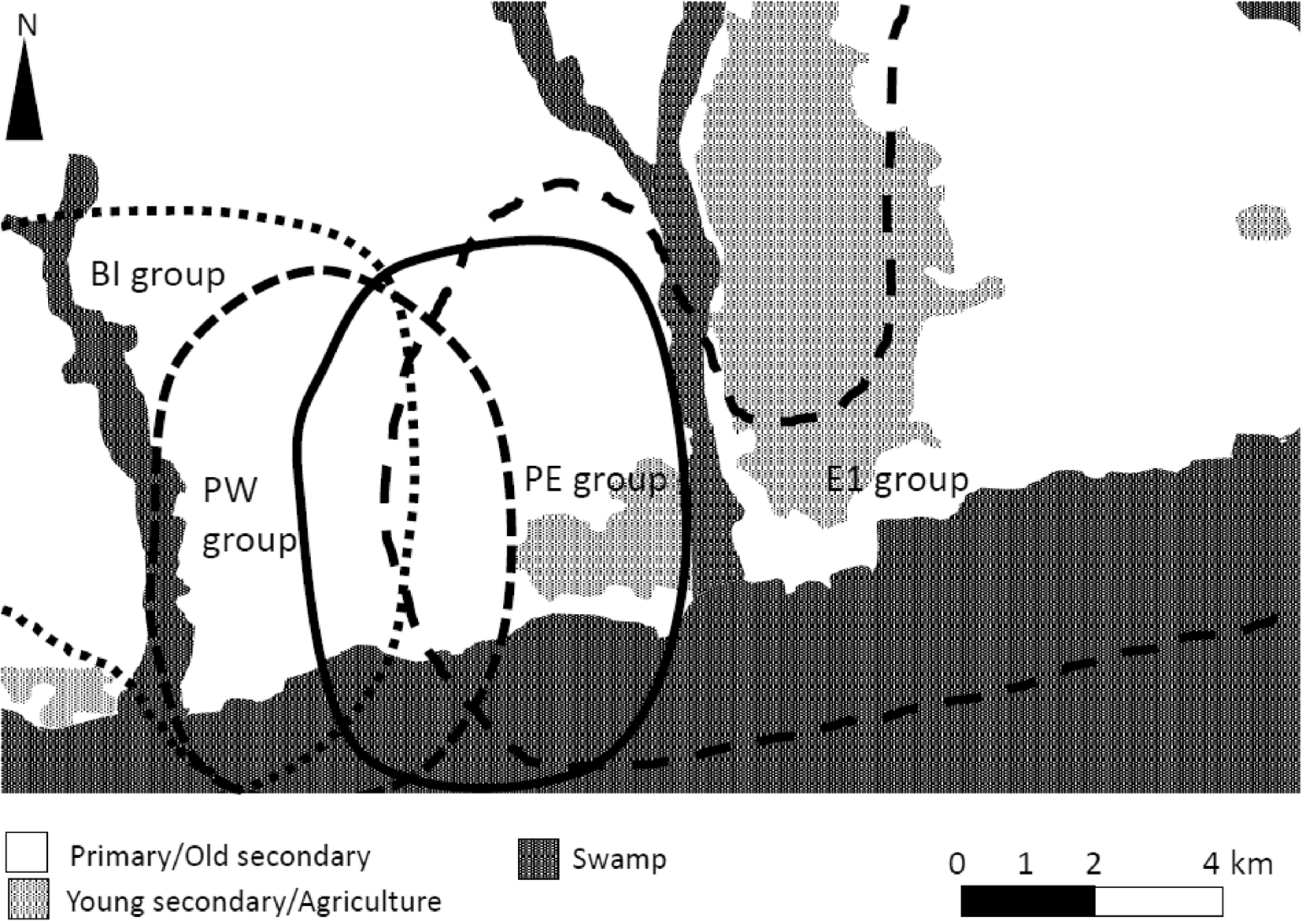
The approximate outlines of the four groups of bonobos that live in the Wamba area (PE, PW, BI and E1)^35^.

Here, we report two cases of a bonobo providing exclusive care for an out-group infant in Wamba, where four groups of bonobos were identified (PE, PW, BI, E1; Fig. 1, see “Methods” for details). We defined “permanent adoption” as provision of species-specific alloparental care by an individual to an immature for longer than a 2-month period, including constant close association, carrying during long-distance travel and nesting together^7,8^. In addition to conducting behavioral observations, we examined the genetic relationship between the adoptive mothers and adoptees.

## Results

### Behavioral observations

Two cases of a female bonobo adopting an infant from different group were observed between April 2019 and March 2020.

#### Case 1: A female with an existing dependent offspring permanently adopted an out-group infant

##### Background

Marie, an adult multiparous female of the PE group, adopted Flora, a female infant from an unknown group. The PE group has been followed daily since 2010. Marie immigrated to the PE group in 2011 and was estimated to be 18 years old in 2019. She gave birth twice, in July 2014 (Marina; female) and March 2017 (Margaux; female), indicating an inter-birth interval (2.7 years) shorter than the average (ca. 4.8 years).

Flora was the daughter of Fula, which was sporadically observed in the BI group between September 2017 and January 2018. Fula was not observed before or after that period, and it was believed that she was temporarily visiting the BI group from an unknown group which was outside of our research area. Although Fula associated with PE individuals for a total of 7 days during group encounters between the PE and BI groups, we did not observe affiliative, agonistic or sexual interactions between Fula and PE individuals. It was unknown whether Fula was alive in April 2019 when Flora was adopted by Marie.

In September 2017, we estimated that Flora was 1.0–1.5 years old. In April 2019, when Flora was observed in the PE group, we estimated that she was 2.6 years old by comparing her size with other infants in the group.

##### Observations

**Figure 2:**
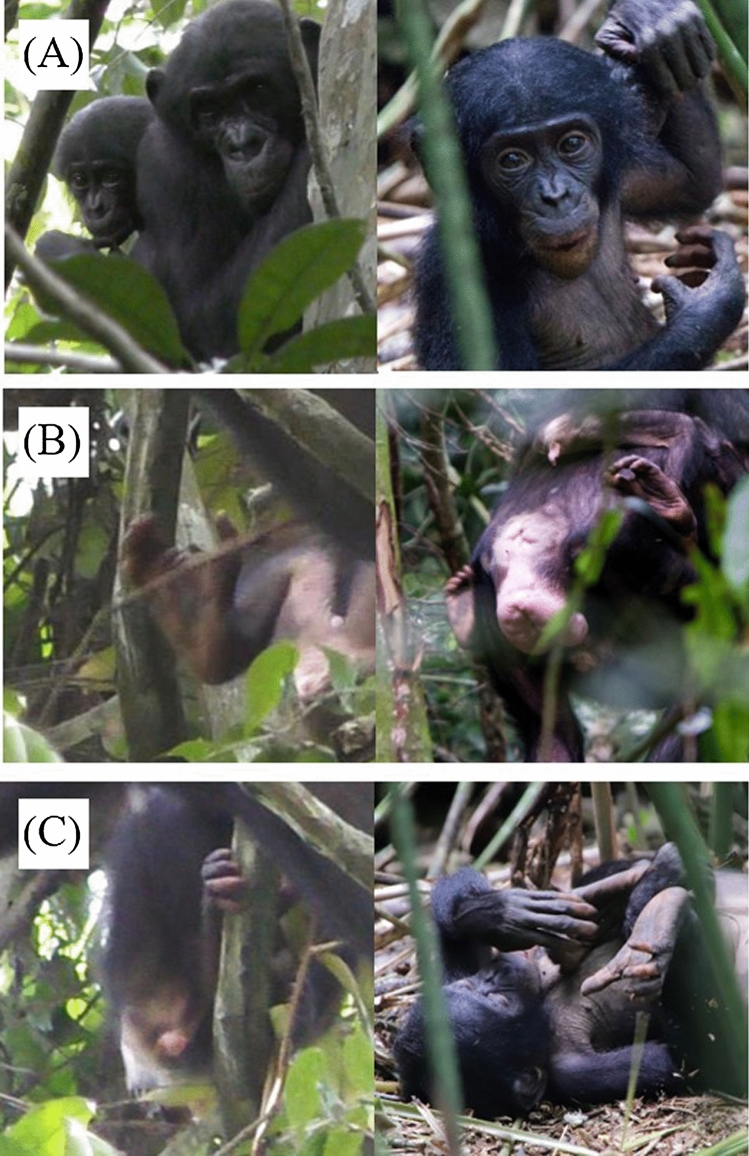
Flora in 2017 (left) and in 2019 (right). (**A**) Facial features include a distinctive U-shaped curve under her nose and raised bumps at the inside corners of her eyebrow ridge. (**B**) Color patterns on the bottom of her right foot, with darker blotches along the inside crease. (**C**) Color patterns of her fingers on her right foot. Her fourth and fifth fingers are pink, and her third finger is black.

Until March 22nd, 2019, we observed Marie carrying two offspring of her own, Marina dorsally and Margaux ventrally. Bonobos were not observed between the 23rd and 29th of March, except for the 26th, but Marie was not seen on that day. We confirmed the presence of an out-group female infant when she was photographed on April 3rd. After carefully examining our photo database, we identified that the infant was Flora, the daughter of Fula (Fig. 2).

**Figure 3:**
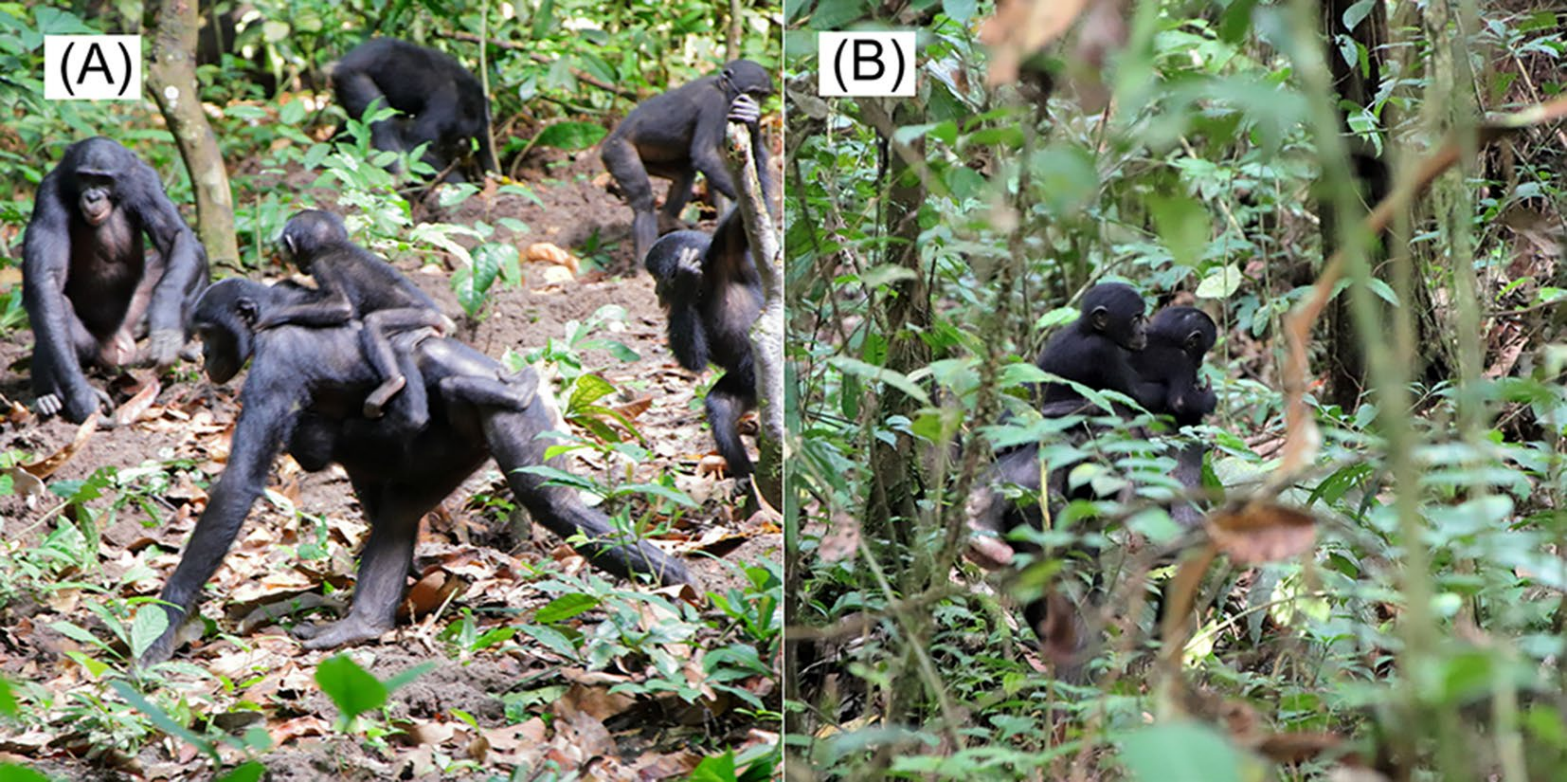
(**A**) Marie carrying Flora ventrally and Margaux dorsally (**B**) Marie carrying both Flora and Margaux on her back.

**Figure 4:**
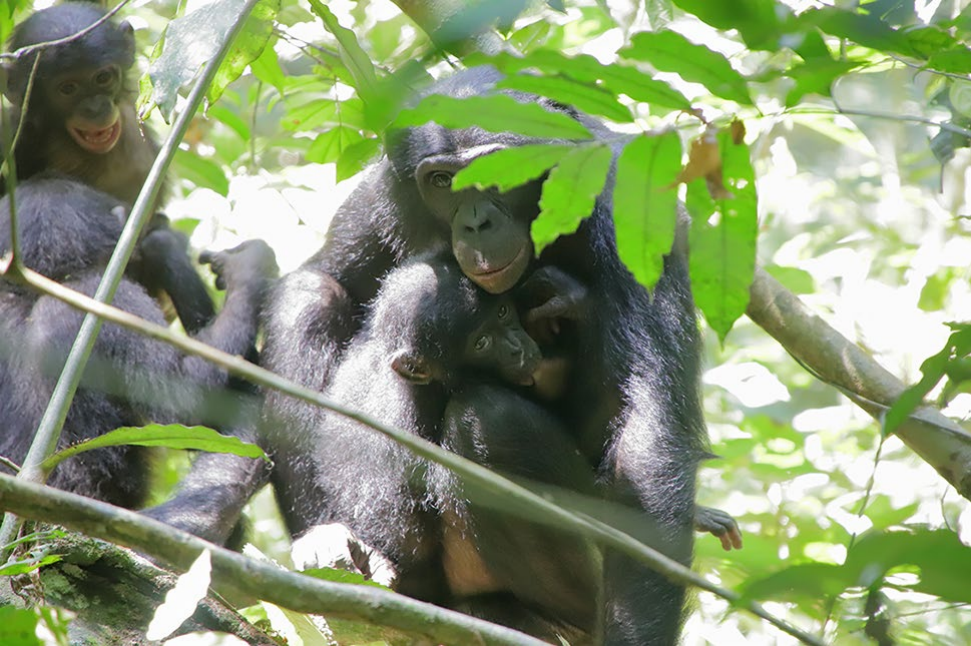
Flora suckling from Marie. Marie’s biological offspring, Marina and Margaux, are visible playing on the left side of the picture.

Marie cared for Flora along with her biological offspring which included constantly staying in close proximity (< 5 m), carrying, grooming, sharing food, nesting together, and nursing. Marie also showed typical maternal comforting behaviours towards Flora: she embraced her or engaged in genito-genital rubbing with her after a distressing episode. Marie either carried Flora ventrally and Margaux dorsally, or carried both infants on her back (Fig. 3) while Marina walked independently. Marina was observed whimpering and screaming when she tried to ride Marie and was rejected twice on April 7th and May 4th. When Marie and Flora initiated travel, we observed typical interactions between mothers and their dependent offspring: Flora ran to Marie, Marie waited for her and then raised her arm to allow Flora to cling. After observing several suspected cases, we confirmed Marie was nursing Flora on April 17th (Fig. 4, Supplementary video S1); Flora was observed suckling from Marie a total of 15 times. We observed Marie sharing food with Flora, Marina and Margaux simultaneously one time (fruit of *Anonidiwn manni*), with Flora and Margaux simultaneously three times (fruit of *Treulia africana* in two cases and young leaves of *Leonardoxa romii* in the other case), with Flora alone once (fruit of *A. manni*) and with Margaux alone once (fruit of *Musanga cecropioides*). Marie groomed Flora and her biological infants in 96 of 1471 (6.5%) total scan data. She directed grooming towards her biological offspring significantly more frequently than towards Flora: of 96 scan data, 13 (13.5%) were directed towards Flora, 30 (31.3%) towards Margaux, and 53 (55.2%) towards Marina (x^2^ = 25.19, p < 0.01, multi-comparison using Ryan’s method: Flora vs Margaux, p = 0.015, α′ = 0.033, Flora vs Marina, p < 0.01, α′ = 0.016, Margaux vs Marina, p < 0.01, α′ = 0.033).

Marina and Margaux never behaved aggressively towards Flora and we often observed play among the three infants (Supplementary video S2). Interactions between Flora and individuals other than Marie and her offspring mostly consisted of play with other immatures. We observed three adult males and one adult female playing with Flora in six different instances; one adult female, an adult male, and three immatures groomed Flora. We never observed aggression towards her from any of the PE group members.

From August 2019, Marie showed behaviors typical of mothers encouraging their offspring to walk independently. For example, she would depart without carrying Flora, but upon Flora's screams she stopped and waited until she climbed on. Although carrying frequency gradually decreased as Flora grew, Marie carried her occasionally and provided other maternal care until the end of our study period (March 2020).

#### Case 2: An old parous female without dependent offspring permanently adopted an out-group infant

##### Background

Case 2 took place in October 2019. An old and parous female of the PW group, Chio, adopted Ruby, a female infant of an unknown group. Although the PW group was not followed daily, bonobos of the PW group were observed through occasional nest-to-nest follows (a few weeks/year) and through inter-group associations with the PE group (see “Methods” for details). Chio and Ruby were observed in the PW group for 57 days between October 2019 and March 2020. Chio was first identified in the PW group in 2012 and had a daughter which emigrated in 2013. Chio was estimated to be 52–57 years old in 2019 and had not given birth since being identified; it was assumed she is post-menopausal. DNA analysis revealed that she has no living offspring in the PW, PE or E1 groups^52^.

Ruby was estimated to be 3.0 years old by comparing her size with other infants in the PW and PE groups when she was adopted by Chio. The natal group of Ruby and her mother remain unknown.

##### Observations

**Figure 5:**
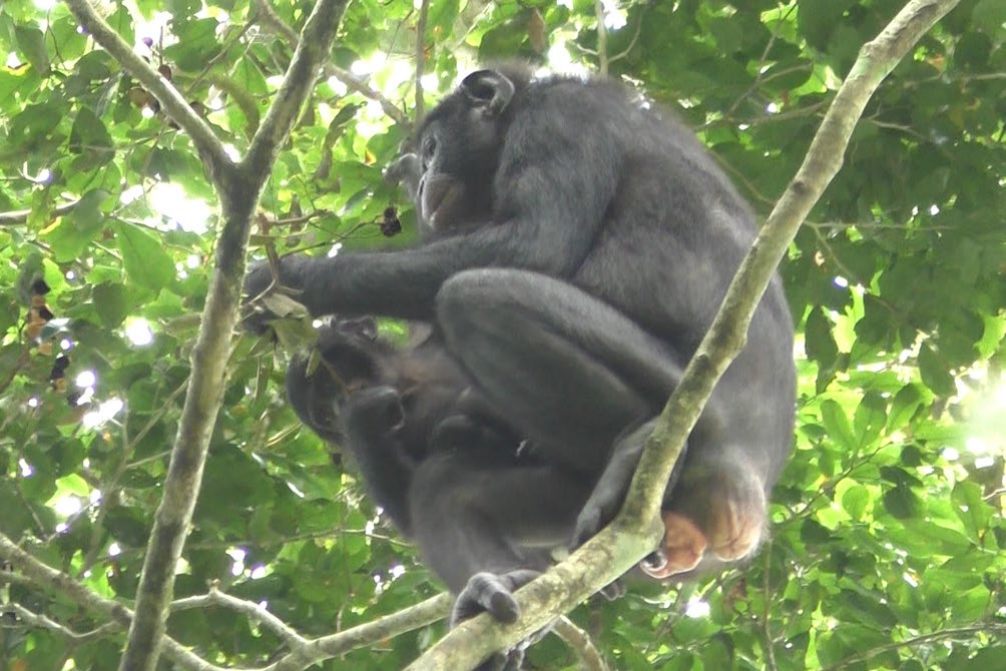
Chio and Ruby feeding on fruit of *Dialium excelsum* from the branch that Chio broke off and was holding.

Chio was observed without an infant during an inter-group association between the PE and PW groups on September 28th, 2019. Four days later on October 2nd, when the two groups associated again, she was carrying an out-group female infant which we later named Ruby. Chio constantly stayed in close proximity to Ruby. She carried, groomed, nested together with, and comforted her by hugging and GG-rubbing throughout the study period (Fig. 5; Supplementary video S3). When Chio initiated travel, she waited for Ruby and carried her either ventrally or dorsally. We also observed Ruby suckling Chio’s nipple once on November 18th. Chio shared food with Ruby twice (fruit of *T. Africana* and *Dialium excelsum*). Although Ruby frequently played with other immatures, we did not observe grooming or any other maternal behaviours towards Ruby from PW and PE adult individuals other than Chio; one exception was when a female juvenile of the PE group briefly carried Ruby on October 2nd. No aggression towards Ruby was observed.

### Kin-relationships between adoptive mothers and adoptees

In both cases, the mtDNA haplotypes of the adoptees was not shared with their adoptive mothers. Additionally, neither adoptee shared a mtDNA haplotype with any other females in their adoptive group.

### Survival of the adoptees after the study period

The observation of bonobos by researchers was interrupted due to the COVID-19 pandemic since the end of the study period (March 2020) until the time of writing (October 2020). Since then, the bonobos have been followed by local assistants and we received a short report about the adoptees twice, in June and October 2020. Flora, the adoptee of case 1 in the PE group, was still well and being cared for by Marie in October 2020. Marie was observed carrying Flora in June 2020 when she was 3.8 years old, but not in October 2020 when she was 4.1 years old. Ruby, the adoptee of case 2 in the PW group, was also well and was still being cared for by Chio, which was observed carrying her both in June and October 2020.

## Discussion

This is the first report of cross-group adoption in great apes: we observed two permanent adoptions by female wild bonobos of an out-group infant (Table 1). The adoptive mothers provided various maternal care to the adoptees, such as transportation, grooming, nursing, and nesting together. Providing protection of an infant during an aggressive episode, which chimpanzee adoptive mothers have done^7,8,15,21^, was not observed in the current cases because there was no aggressive behavior by members of the adoptive group towards the adoptees.

**Table 1:** The information of adoptees, biological and adoptive mothers, and duration of adoption.

	Adoptee (age, natal group)	Biological mother (age, group)	Adoptive mother (age, group)	Occurrence of adoption	Survival of the adoptee in October 2020 (duration of the adoption)
Case 1	Flora (2.6, unknown)	Fula (30–35, unknown)	Marie (19, PE)	April 2019	Yes (18 months)
Case 2	Ruby (3.0, unknown)	Unknown	Chio (52–57, PW)	October 2019	Yes (12 months)

Although bonobo groups sometimes encounter each other and associate for a period consisting of a few hours up to days, they usually separate back into their original group when the inter-group association ends, except for young nulliparous females. Playback experiments in captive bonobos confirmed that they have long-term memory and can distinguish group members from out-group individuals^53^. Considering their stable group membership and capacity of individual recognition, it is highly unlikely that the cross-group adoptions were accepted due to the vagueness of group member identification skills as suggested in titi monkeys^30^. Although bonobos likely recognized that the adoptees were from a different social group, they accepted the infants and did not display aggression towards them. In bonobos, infanticide or severe aggression towards within- or out-group infants has never been reported^38,39^. During inter-group associations, infants often play with and are groomed by individuals of other groups, including adult males (N. Tokuyama, personal observation). Although this is the first reported case of an infant staying alone in a different social group, other cases of temporary or long-term stay in an out-group exist: an adult female and male stayed in a large party of a neighboring group and received only mild aggression^35,37,54^, a fragmented group fused with a neighboring group^56^, and resident females often tolerated newly immigrated females^57^. In addition to observations in the wild, bonobos in captivity proactively shared food during behavioral experiments with individuals from different social groups^58,59^. Also, the introduction of immature or mature bonobos to an unfamiliar group in zoos and sanctuaries usually proceeds relatively smoothly compared to that of chimpanzees^60,61^. The cases of cross-group adoptions presented here are additional examples of high social tolerance among bonobos, both towards out-group and immature individuals.

In the current cases, an estimated 2.6 year old (case 1) and 3.0 year old (case 2) infant without a biological mother were adopted and have so far survived > 18 months and > 12 months, respectively. Both infants were females and younger than 5.0 years old at the time of adoption, a period when a mother’s care is crucial for an infant’s survival^43,44^. Both adoptees suckled from their adoptive mothers, although it would be necessary to analyze the stable isotope or breast milk protein in the adoptees’ fecal samples to confirm whether they consumed milk or if such nipple contact was “comfort nursing” (no milk consumption). Previous studies using fecal and dental stable isotopes showed that chimpanzee infants’ reliance on breast milk begins to decline from 1.0 year (Ngogo) or 2.0 years (Taï) of age, but that they typically continue to consume milk until they complete weaning at the age of 4.0–4.5^62,63^. Considering this along with the fact that the adoptive mother in case 1 had a 2.1 year old infant of her own, it is likely that she was lactating and that this nutritional benefit may have contributed to the survival of the adoptee. In case 2, the adoptive mother was presumed to be post-menopausal and may not have been lactating, so the infant may have made nipple contact for comfort rather than for receiving milk^63^. In both cases, the adoptees may have benefited from their adoptive mothers which provided transportation and grooming, as well as emotional support and social learning opportunities^45–47,64,65^.

Adoption might incur considerable costs to adoptive mothers. The adoptive mother in case 1, Marie, might have experienced particularly high costs because she already had two dependent biological offspring. She nursed and carried the adoptee and her younger offspring (2.1 years old) simultaneously, and we also sometimes saw her older offspring (4.8 years old) suckle. She spent time grooming all three young. In great apes, mothers do not usually resume reproduction until their offspring are weaned, thus rearing multiple unweaned infants is considered to be costly. However, individuals in a good health condition tend to have a shorter interbirth interval^66^. Due to her unusually short inter-birth interval (2.7 years, average is 4.8 years), Marie had already experienced rearing two biological infants simultaneously, including “tandem-breastfeeding” (T. Sakamaki, personal observation). Her good physical condition may have enabled her to adopt the third infant. Our observation suggests that Marie did not provide equal maternal care towards the adoptee and her biological offspring. She groomed her biological offspring more frequently than the adoptee, and her older biological offspring showed signs of distress when her mother stopped carrying her due to the presence of the adoptee, suggesting there may have been some conflict. However, we did not observe any aggression or clear rejection from the adoptive mother or her biological offspring towards the adoptee. In August 2019, the adoptive mother first rejected to carry the adoptee (2.9 years old at that time), which is a typical behavior of mothers encouraging their offspring to walk independently.

Kin selection is considered to be the primary mechanism for the evolution of adoption^5,9,16,20^. Bonobos are a female dispersing species, so females may have close female kin residing in their neighboring groups^52,67^. However, in the current two cases of cross-group adoption, the adoptive mothers and adoptees did not share a mtDNA haplotype, suggesting that they were not maternally related to each other. Although it is still possible that the adoptive and biological mothers are paternally related each other, paternal relatedness might not be the direct motivation of these adoption cases due to bonobos’ promiscuous mating patterns. In chimpanzees, orphans have been adopted by their maternal older siblings, but not by their paternal siblings^8^. This may be because kin recognition among paternal siblings is less developed compared to among maternal siblings due to their promiscuous mating pattern^68^.

Another potential mechanism for adoption behavior may be that it provides a direct fitness benefit to adoptive mothers through the improvement and development of parenting skills and social allies^5,7,18,20^. However, the adoptive mothers in these cases may not have needed additional practice for future parenting as the adoptive mother in case 1 already had two dependent offspring, and the adoptive mother in case 2 was presumably post-menopausal. One possibility is that adoptees could become future allies of the adoptive mothers. Both adoptees were females, and female bonobos form strong social bonds and coalitions within their group and sometimes across groups^37,69–72^. Although female adoptees are likely to emigrate from the group upon maturation, females in this area tend to emigrate into neighboring groups and so the opportunity to maintain social relationships through inter-group associations exists^52,73^. Thus, it is possible that future social bonding and alliances would be beneficial for the adoptive mothers. Additionally, it is possible that the adoption of an infant may improve the current social relationships of an adoptive mother in the group; infants attract other young females^18^, so it may provide opportunities to reinforce social bonds with other females. This may partly explain why the adoptive mother in case 2 which was old and did not have her own offspring adopted an infant, although we were not able to analyze her social relationships before and after the adoption because her group was not the main subject of our daily follows.

Pre-existing social relationships between biological and adoptive mothers promote adoption in chimpanzees and humans^7,21^. Chimpanzee orphans are primarily adopted by maternal siblings, which may be due to strong social relationships rather than genetics^8^. When an orphan does not have a maternal sibling in the group, he/she is often adopted by unrelated individuals which had a close social relationship with the deceased mother^7,21^. However, when biological and adoptive chimpanzee mothers belong to different social groups, no pre-existing social relationship may exist. Unlike chimpanzees, bonobos living in different social groups, especially females, associate, interact, and even cooperate^34,35,37,74^. Despite this, in case 1 we did not observe affiliative, sexual, or aggressive interactions between the biological mother and individuals in the adoptive group. We were unaware of the relationship between the biological and adoptive mother in case 2 because we could not identify the biological mother. Although we cannot rule out the role of pre-existing social relationships in the occurrence of adoption, it did not appear to be essential, at least in case 1.

Although adoption in humans could have adaptive functions^22,24,75,76^, it is often motivated by factors such as empathy, a drive to contribute to society, and/or a strong desire to raise a child, i.e. the by-product of other evolutionary adaptations^22,23,76^. Chimpanzees are also known to adopt infants without an apparent direct or indirect benefit; adoption may not be entirely for their own benefit, but could be driven by altruism and emotion^7,21^. Similarly to humans and chimpanzees, adoption in bonobos may be triggered by emotion, altruism, and/or their strong attraction to infants. Bonobos react emotionally to other individuals’ pain and dissatisfaction, for example they give attention to emotional scenes, try to help injured individuals, and comfort distressed individuals^46,77–80^. Additionally, females are strongly attracted by infants and kidnapping sometimes occurs^18,81–84^. It might also be noteworthy that we observed Chio, the adoptive mother in case 2 which did not have a dependent offspring for 6 years, temporarily providing intensive alloparental care such as carrying and grooming to two infants (separately) in 2018 and 2019 which were not well taken care of at the time due to the poor health of their mothers (Tokuyama, personal observations). The latter case was observed four months before she adopted Ruby. Also, Chio carried the dead body of a red tailed monkey for over a month in 2016^85^, which could suggest that she has a tendency of being attracted towards and providing care to infants.

## Conclusion

Adoption in wild bonobos goes beyond the boundaries of social groups and is not necessarily related to kin relationships or to pre-existing social relationships between adoptive and biological mothers. The current cases of cross-group adoption may have been enabled or driven by bonobos’ altruism, strong attraction to infants, and/or high social tolerance towards out-group individuals. Recognizing this may contribute to a better understanding of adoption in humans.

## Methods

The two cases presented in this study were observed between April 2019 and March 2020 in the northern part of Luo Scientific Reserve (Wamba), the Democratic Republic of the Congo, where the long-term study of wild bonobos has been conducted since 1973^86^. After this study period, observations by researchers were interrupted due to the COVID-19 pandemic until the time of writing. Since then, the bonobos have been followed by local assistants and we received a short report about the study subjects in June and October 2020.

Four wild bonobo groups (PE, PW, BI, and E1) inhabited the area during the study period, two of which (PE and PW) were of focus in this study^35^ (Fig. 1). The PE and E1 groups have been followed daily since 2010 and 1973, respectively. The PW and BI groups have been followed less frequently, a few times (a few days or weeks) per year, and observed during inter-group encounters and associations with the PE group. All individuals of the PE, PW, and E1 groups have been identified. All adult individuals of the BI group were identified by the start of this study, but the identification of subadults and immatures was ongoing. During the study period, the nest-to-nest observations of the PW group was conducted between November 18th and 25th, 2019. The PE group consisted of 3–4 adult males, 8 adult females, 0–2 immigrant females, and 12–14 immatures. The PW group consisted of 4 adult males, 7 adult females, 0–2 immigrant females, and 4–5 immatures.

### Behavioral observation

Following Boesch et al. and Hobaiter et al., we considered the provisioning of species-specific alloparental care by an individual to an immature for longer than a 2-month period as “permanent adoption”, including constant close association, carrying during long-distance travel and nesting together^7,8^. Observations were made during each researcher’s own projects. In case 1, the grooming behavior of the adoptive mother was recorded using instantaneous scan sampling at 5-min intervals. We recorded other maternal behaviors that the adoptive mother provided to the adoptee ad libitum, such as carrying, nursing, food sharing and genito-genital rubbing. In case 2, all behaviors between the adoptive mother and adoptee were recorded ad libitum. In both cases, we recorded the social interactions between the adoptees and group members other than their adoptive mothers ad libitum.

### Genetic analysis

We investigated the genetic relationship between adoptive mothers and adoptees by collecting non-invasive fecal DNA samples. Mitochondrial haplotypes of the two adopted infants were newly determined. Following the methods adopted in our previous work^52^, we analyzed the nucleotide sequence for the 915-bp portion of the mtDNA control region, including the hypervariable region I and II. The nucleotide sequence was analyzed twice or more to confirm the accuracy of the results. Based on the nucleotide sequence, mtDNA haplotypes for the infants were determined. To avoid any confusion of the sample identification, at least two independently collected samples were used for the analysis when possible; however, we were only able to collect one sample from Flora (case 1).

The mtDNA haplotypes of the adult females of the three groups (E1, PE, and PW) were analyzed in our previous work^52^. Using this existing data, we examined whether the mtDNA haplotype of each adopted infant was shared with its adoptive mother, and if it was not shared, we concluded that there was no matrilineal kinship between them.

### Ethical note

The wild bonobos were habituated and we observed and collected samples without the use of invasive methods. This study complied with the Guideline for Field Research of Non-human Primates of the Primate Research Institute of Kyoto University (https://www.pri.kyoto-u.ac.jp/research/Guideline%20for%20field%20research%20of%20non-human%20primates201905.pdf), and the ARRIVE guidelines, as well as the legal requirements, including research permission, of the Democratic Republic of the Congo. This study was approved by the Research Centre for Ecology and Forestry, and the Ministry of Scientific Research of the Democratic Republic of the Congo.

## Supplementary Information

Supplementary Legends.

Supplementary Video S1.

Supplementary Video S2.

Supplementary Video S3.

## Data Availability

The observational and genetic data are available from the corresponding author on reasonable request.
