# Supplementary material for: Two wild female bonobos adopted infants from a different social group at Wamba
Source: Sci Rep. 2021 Mar 18;11:4967. doi: 10.1038/s41598-021-83667-2 (PMC7973529; doi:10.1038/s41598-021-83667-2)
Supplement: Supplementary file 1 — Supplementary Legends. [file 41598_2021_83667_MOESM1_ESM.docx]

Supplementary material

**Two wild female bonobos adopted infants from a different social group at Wamba**

Nahoko Tokuyama^1,2^, Kazuya Toda^1^, Marie-Laure Poiret^3^, Iyokango Bahanande^4^, Batuafe Bakaa^4^, Shintaro Ishizuka^1^

^1^ Primate Research Institute, Kyoto University, Kanrin 41, Inuyama, Aichi 484-8506, Japan

^2^ Wildlife Research Center, Kyoto University, Japan

^3^ Department of Psychology, Durham University, United Kingdom

^4^ Research Center for Ecology and Forestry, Democratic Republic of the Congo

Supplementary video legends

Supplementary video 1. Flora suckling from Marie while Marie feeds on truffles. Margaux was on Marina’s back.

Supplementary video 2. Flora and Margaux playing while Marie grooms Marina.

Supplementary video 3. Chio holding Ruby and feeding on the fruit of *Dialium excelsum*. Ruby is feeding on the fruit from the branch that Chio broke off and is holding. When Chio starts moving, the branch with fruits is transferred from Chio to Ruby.
